# How ‘place’ matters for addressing the HIV epidemic: evidence from the HPTN 071 (PopART) cluster-randomised controlled trial in Zambia and South Africa

**DOI:** 10.1186/s13063-021-05198-5

**Published:** 2021-04-06

**Authors:** Virginia Bond, Graeme Hoddinott, Lario Viljoen, Fredrick Ngwenya, Melvin Simuyaba, Bwalya Chiti, Rhoda Ndubani, Nozizwe Makola, Deborah Donnell, Ab Schaap, Sian Floyd, James Hargreaves, Kwame Shanaube, Sarah Fidler, Peter Bock, Helen Ayles, Richard Hayes, Musonda Simwinga, Janet Seeley, on behalf of the HPTN071 (PopART) study team

**Affiliations:** 1 Department of Global Health and Development, Faculty of Public Health and Policy, London School of Hygiene and Tropical Medicine (LSHTM), 15-17 Tavistock Place, London, WC1H 9SH UK; 2 Zambart, School of Public Health, University of Zambia, Ridgeway Campus, P.O. Box 50697, Lusaka, Zambia; 3 Desmond Tutu TB Centre, Department of Paediatrics and Child Health, Faculty of Medicine and Health Sciences, Stellenbosch University, P.O. Box 241, Cape Town, 8000 South Africa; 4 Fred Hutchinson Cancer Research Center, 1100 Fairview Ave. N., P.O. Box 19024, Seattle, WA 98109-1024 USA; 5 Department of Infectious Disease Epidemiology, Faculty of Epidemiology and Population Health, LSHTM, Keppel Street, London, WC17HT UK; 6 Centre for Evaluation, Faculty of Public Health and Policy, LSHTM, Keppel Street, London, WC17HT UK; 7 National Institute for Health Research Biomedical Research Centre, Imperial College, South Kensington, London, SW7 2BU UK; 8 Department of Clinical Research, Faculty of Infectious and Tropical Diseases, LSHTM, Keppel Street, London, WC17HT UK; 9 Africa Health Research Institute, Nelson R. Mandela Medical School, 719 Umbilo Rd, Durban, 4001 South Africa

**Keywords:** Community randomised trials (CRTs), Social context, Southern Africa, Communities, Stability

## Abstract

**Background:**

In a cluster-randomised trial (CRT) of combination HIV prevention (HPTN 071 (PopART)) in 12 Zambian communities and nine South African communities, carried out from 2012 to 2018, the intervention arm A that offered HIV treatment irrespective of CD4 count did not have a significant impact on population level HIV incidence. Intervention arm B, where HIV incidence was reduced by 30%, followed national guidelines that mid trial (2016) changed from starting HIV treatment according to a CD4 threshold of 500 to universal treatment. Using social science data on the 21 communities, we consider how place (community context) might have influenced the primary outcome result.

**Methods:**

A social science component documented longitudinally the context of trial communities. Data were collected through rapid qualitative assessment, interviews, group discussions and observations. There were a total of 1547 participants and 1127 observations. Using these data, literature and a series of qualitative analysis steps, we identified key community characteristics of relevance to HIV and triangulated these with HIV community level incidence.

**Results:**

Two interdependent social factors were relevant to communities’ capability to manage HIV: stability/instability and responsiveness/resistance. Key components of stability were social cohesion; limited social change; a vibrant local economy; better health, education and recreational services; strong institutional presence; established middle-class residents; predictable mobility; and less poverty and crime. Key components of responsiveness were community leadership being open to change, stronger history of HIV initiatives, willingness to take up HIV services, less HIV-related stigma and a supported and enterprising youth population. There was a clear pattern of social factors across arms. Intervention arm A communities were notably more resistant and unstable. Intervention arm B communities were overall more responsive and stable.

**Conclusions:**

In the specific case of the dissonant primary outcome results from the HPTN 071 (PopART) trial, the chance allocation of less stable, less responsive communities to arm A compared to arm B may explain some of the apparently smaller impact of the intervention in arm A. Stability and responsiveness appear to be two key social factors that may be relevant to secular trends in HIV incidence. We advocate for a systematic approach, using these factors as a framework, to community context in CRTs and monitoring HIV prevention efforts.

**Trial registration:**

ClinicalTrials.gov NCT01900977. Registered on July 17, 2013.

**Supplementary Information:**

The online version contains supplementary material available at 10.1186/s13063-021-05198-5.

## Introduction

Community-based service delivery is critical for extending the reach of HIV prevention to address the high burden of HIV in sub-Saharan Africa [1]. A community-based approach is embedded in the universal testing and treatment (UTT) strategy that was evaluated in four population-based cluster-randomised trials (CRT) in Africa [2–5]. Findings from the largest of these, HPTN 071 (PopART)) in Zambia and South Africa, were published in 2019 [2]. The trial had a somewhat puzzling primary outcome result. Communities where combination HIV prevention (including UTT) linked recipients to anti-retroviral treatment (ART) from the start of the trial, irrespective of CD4 count and prior to changes in national recommendations (arm A of the trial), performed less well in reducing HIV incidence than those where ART was commenced in accordance with national guidelines. It should be noted that national guidelines changed to universal treatment for all people living with HIV half way through the trial (2016) [2]. Further there was significant overlap in confidence intervals to incidence point estimates meaning that while the overall trend is robust, apparent community-level ‘differences’ are easily over-interpreted. The initial conclusion reached by the trial team was that the dissonant difference between intervention packages may have been due to chance, pending further interdisciplinary analysis [2]. The social science analysis presented here is responding to a broad interest in understanding what might have contributed to the unexpected primary outcome results. Our focus is on community characteristics during the trial (2013–2018) as impacting the HIV epidemic trajectory at community level, rather than an evaluation of how community characteristics influenced the trial intervention.

In some CRT research [6], there is mention of the importance of ‘real world settings’. However, there is often no detail on or discussion of distinct communities/places and differences between communities involved in the trials, with a few exceptions [7]. The primary outcome results from the four UTT trials in sub-Saharan Africa convey community variability in the range of HIV incidence point estimates by community, and by indicating the degree of certainty in the primary outcome [2–5, 8]. However, links between social context and HIV incidence have not yet been detailed. There are calls to be more explicit about the complexity and variability of trial settings [9, 10] and to provide more detail about the interaction between context and the intervention in the interpretation of primary and secondary outcomes [10–15]. The HPTN 071 (PopART) trial provided an unusual opportunity to combine methods and disciplines and to investigate the value of making community context more apparent.

We used the dissonant finding to reflect on community secular influences on incidence that complicate the planning, implementation and interpretation of data from community-randomised trials. Data available for this analysis were extensive, from multiple sources before and during the trial period, and built on social theory of urban communities and social factors of significance to health and HIV [16–18].

## Methods

### HPTN071 (PopART) design

The aim of the HPTN 071 (PopART) trial was to evaluate the impact of the PopART combination HIV prevention intervention package on HIV incidence at population level [19]. HPTN 071 (PopART) was carried out in 21 Zambian and South African urban communities from 2012 to 2018 [2, 19]. The study communities were selected based on HIV burden (relatively high prevalence), geographical location and stakeholder support and approval [19]. Communities were defined as the catchment area population of a health facility delivering ART. Population size varied from 18,000 to over 100,000. Community engagement processes played a pivotal role in ethical practice and communication about the trial research and intervention [20]. The trial had two intervention arms (labelled ‘A’ and ‘B’) and one control arm (labelled ‘C’). Study communities were randomly allocated to arms within seven triplets matched on HIV prevalence estimated at baseline, geographic and demographic profile [19]. The PopART arms were well balanced, matching factors considered epidemiologically to be important to HIV incidence [2]. The intervention was an HIV combination prevention package including UTT. In arm A, trial-employed community health workers encouraged the uptake of ART regardless of CD4 count, whereas in arm B, the national guidelines, which changed over the course of the trial and by mid-way through included ART regardless of CD4 count, prevailed [19]. The primary outcome, HIV incidence between 12 and 36 months, was measured through a population cohort of randomly selected adults [18–44] followed up for three years. Consenting participants had their blood collected and tested for HIV using laboratory-based tests at each of four rounds over the 3 years.

### Social science component of the trial

From the outset of the trial, the social science component was intended to document similarities and differences across the 21 study communities and to evaluate what differences were relevant to the uptake of HIV prevention options [21, 22]. Therefore, we collected data systematically on social context from all communities prior to the intervention, during the intervention and for a short period after the end of the intervention. From the beginning, the social science design used a typology model developed out of research in urban communities in Europe and Africa to manage, compare and communicate the complexity of urban communities through documenting key salient indicators [16, 23, 24]. The approach for assessing the intervention implementation was labelled ‘Story of the Trial’ and drew on a process evaluation approach [25] to investigate the implementation of the intervention, research and community engagement. In addition to the core social science design, ancillary studies carried out on stigma [26] and young people [27] led to supplementary community level data. These qualitative data sets collectively stretch from 2012 to 2018 and are summarised in Table 1 and described in more detail in Additional File 1.

**Table 1:** Qualitative data sources

Core qualitative activities across the 21 HPTN 071 (PopART) communities			
Data source	Description	Data for analysis	Timeframe
Broad Brush Surveys (BBS): Formative Research	Rapid, qualitative, participatory survey approach in each community prior to PopART implementation to gauge relevance of physical features, social organisation, networks and community narratives for HIV. Group discussions, structured observations and interviews.	Group discussions: 129 Key informant interviews: 95 Participants: 1202 (744 women) Observations: 203	2012–2013
PopART Social Science Story of the Trial	Qualitative documentation of intervention and research implementation, community engagement and community response throughout intervention period. Observations, group discussions and in-depth interviews.	Observations: 763 Group discussions: 24 In-depth interviews: 36 Participants: 263 (147 women)	2014–2018
P-ART-Y (PopART for Young People)	Mapping and observing services and spaces for young people (aged 10–24) in PopART communities. Structured observations prior to and during the P-ART-Y intervention that included informal discussions with young people and a qualitative stakeholder survey.	Observations: 161 Stakeholders: 82	2015–2017

### Qualitative data teams, collection and management

Social science teams in both countries consisted of experienced social scientists, including graduates and research assistants with extensive field-based knowledge. The teams received the same training for distinct phases or ancillary studies through collaborative workshops held in either Zambia or South Africa. Social scientists within the team often carried out the different research activities sequentially in particular communities, from 2012 to 2018, building up their understanding, familiarity and rapport with these communities. The same research activities and instruments, data capture tools, data management system and data quality assurance system were used across countries. Rapid analysis for trial needs preceded more focused, coded analyses for all three data sets (BBS, Story of the Trial, P-ART-Y). Rapid analysis summaries were repeatedly cross checked with research teams across countries and refined accordingly. Regular debriefing of in-country teams (in groups and one-on-one) and across countries, refresher training, data analysis workshops and discussion of concerns, key findings and events were held. See Additional File 1 for detail.

### Qualitative data analysis steps

**Fig. 1:**
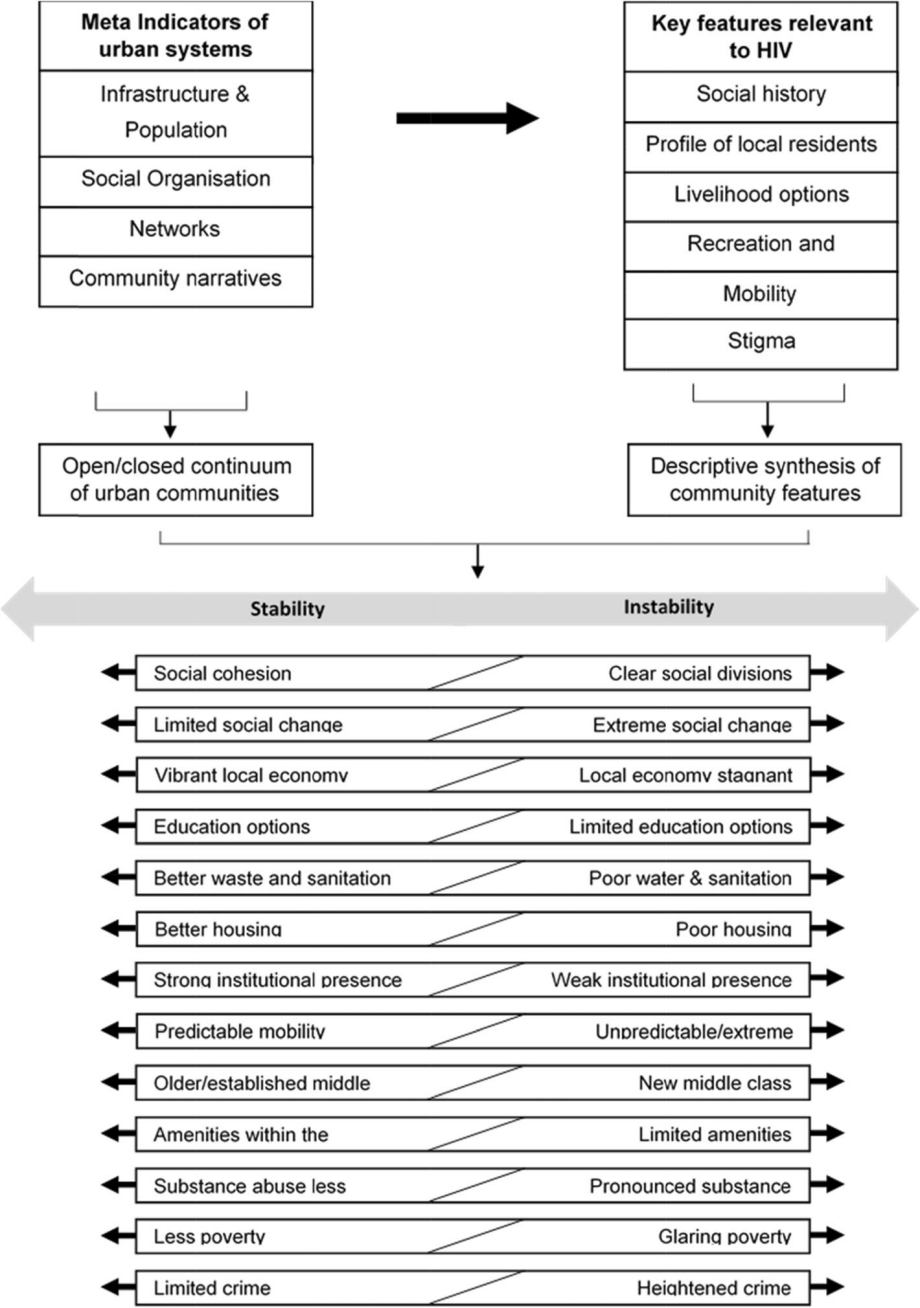
Process of synthesising qualitative community level data to identify stability/instability

**Fig. 2:**
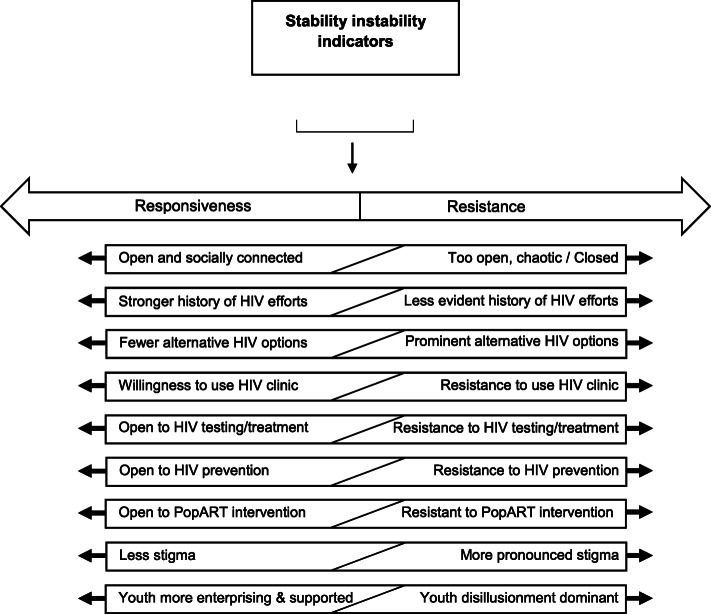
Responsive/resistant social factor

Identifying the influence of community context on HIV meant generating a synthesis of this comparative community level data. The earlier analysis steps were conducted before the unblinding of the primary outcome results, and later steps after the unblinding, with checks in place to address bias. These checks were independent reviews of community data by key researchers and not triangulating the social factors with HIV incidence across all communities until the community level analysis steps were completed. In brief, we first summarised meta-indicators of each urban community and then drew on these data to summarise six key features. These features represent our synthesis of existing HIV literature, our own analysis and urban systems theory interpreted and extended through our data [17, 18, 23, 28, 29]. We then identified the encompassing social factor stability/instability and then, to more specifically address HIV, the interdependent social factor responsiveness/resistant. Figures 1 and 2 detail the sequence of analysis, with additional explanatory detail in Additional File 1.

The lead author then generated a summary table of all communities that were checked by other co-authors. We then considered the distribution of the two social factors across communities ‘types’ relative to HIV incidence and trial arm to see if a pattern emerged.

### Ethical approval

The study, including all sub-components and ancillaries, was approved by Research Ethics Committees (REC) at the London School of Hygiene and Tropical Medicine, University of Zambia, and Stellenbosch University. We also received approval from the Ministry of Health in Zambia and the Department of Health (Western Cape Province and Cape Metropolitan District) in South Africa. All study participants who were formally interviewed (qualitatively) or surveyed (quantitatively) provided written informed consent per the local REC guidance. Qualitative structured observations obtained verbal consent from settings (for example, the health facility and households) and community leaders. We use trial codes (the first letter of codes: Z = Zambia, SA = South Africa) to protect confidentiality.

## Findings

## Historical trajectories of communities and HIV

In 2013, all Zambian trial communities were well-established urban communities, most dating back to the 1960s, but five of the nine South African trial communities were relatively new, developing from the mid-1990s onwards. Some underwent rapid social change either just prior to the trial or during the trial. In South Africa, for example, SA14 had a prior history of extreme social change from an informal settlement to a planned neighbourhood with formal housing. During the trial, five communities had geographically expanding boundaries and two South African communities had very fast-growing informal settlements. Another trend in all South African communities was the continued emergence of shack dwellings in the yards of Reconstruction and Development Programme (RDP) housing (government subsidised housing), pushing up population numbers and density. In Zambia, there was a pattern of new middle-class residents moving into six communities, often with few commitments to the local community, and accessing employment, school and sometimes private health care outside of the community. Disruptive events during the trial were other types of change. These included a cholera outbreak in three Zambian communities in 2017–2018, flooding leading to displacement in a South African community in 2016 and political protests about services and arson in another in 2017–2018. Only in five communities (Z1, Z2, Z3, SA20, SA21) was there much more limited social change.

The influence of place on HIV prevalence prior to the trial emerges through grouping the communities by provincial and town location in Zambia and by location within or close to a city in South Africa. At country level, HIV prevalence varied across communities. In Zambia, HIV prevalence was in a similar range at provincial level across communities; the range was smaller at the level of a district town and slightly wider in a large capital city. In South Africa, HIV prevalence was in a similar range for communities that were clustered close together within the same geographical area both within the city and outside. For a group of communities (SA16 to SA19) spread across the city and not clustered together, the HIV prevalence range was considerably wider. Table 2 presents the HIV prevalence of trial communities in 2013-2015 derived from trial data from the population cohort.

**Table 2:** Community HIV prevalence at the beginning of HPTN071 (PopART)

	Community code	PC0 prevalence (2013–2015) (%)	Geographical location
Triplet 1	Z1	17	Province 1, district town 1
	Z2	16.3	
Triplet 2	Z4	18.1	Province 1, district town 2
	Z5	17.4	
Triplet 1	Z3	21.9	Province 2, district town
Triplet 2	Z6	23.4	
Triplet 3	Z7	19.6	Province 3, capital city
	Z8	18.3	
	Z9	21.4	
Triplet 4	Z10	25.9	Province 4, district town 1
	Z11	24.2	
	Z12	28.1	Province 4, district town 2
Triplet 5	SA13	29.2	City Zone 1
	SA14	29.9	
	SA15	28	
Triplet 6	SA16	24.9	City Zone 2
	SA17	35.7	City Zone 3
	SA18	19.9	City Zone 4
Triplet 7	SA19	10.8	Municipal area 1 outside city
	SA20	9.9	
	SA21	2.99	Municipal area 2 outside city

## Stability and instability: encompassing social factor

In our data, stability was influenced a variety of factors that were more or less present in the 21 communities. These factors include the following: social cohesion, social change, economic opportunity, amenities, mobility, class profile, substance use, crime and poverty. Examples are provided in Additional File 2.

### Social cohesion

Social cohesion manifested in an established and social connected community that arose from longer term residents identifying with the place, a generational (and thus age) mix, a class, ethnic and wealth mix that was either more homogenous or had an established history of diversity and a strong shared history. For example, 10 of the 21 communities had stable core areas with families who have been there for generations. Some communities were considered nice places to live, offering security, infrastructure and residents who could rely on each other. In contrast, social divisions manifested in class and wealth divisions, racial and ethnic divisions, divisions between older and newer residents, a strong population age imbalance and a limited shared history.

### Social change

Not being open to change and extreme social change either current or in the recent past was destabilising. Extreme social change was characterised by new areas emerging with expanding boundaries or rapid population influx, as described earlier, or with a new class of residents moving in.

### Economic options

A vibrant local economy was demonstrated by wider and stronger employment options (either a mix of formal and informal, or a robust informal economy) and a busy market located within the community that draws people into the community and provides trade and work. A stagnant local economy was characterised by limited employment and livelihood options (both limited formal employment and limited and precarious informal) and by being empty in the day, with people leaving the community to earn a living outside because the local economy offered very limited options.

### Local amenities—education, health, water and sanitation, housing, institutional presence

Education options were manifested in the presence or absence of secondary schools within the communities. Whilst all communities had primary schools and most had a secondary school (a few Zambian communities did not have secondary schools which led to secondary school children travelling out of the community every day). Overall, the more access to amenities like libraries, sport, and trading areas, the more stable the communities. More amenities were better for community members providing constructive outlets and boosting community identity. Housing acted as an indicator of history, socio-economic class and social change and often varied within communities in house size and quality. The RDP transformed the landscape of some South African communities prior to or during the trial, replacing areas of shacks with improved housing structures. Stronger institutional presence came in the form of political affiliations (with, for example, the ruling party), historical links with mines and councils and the presence of prisons, the army, police, church and key non-governmental organisations.

### Mobility

There was predictable mobility characterised by daily movement linked to livelihoods (for example, travelling to markets to buy and sell or travelling to farms), transport hubs and education and seasonal mobility. In South Africa, the latter was linked to farming and holidays in the Eastern Cape, and in Zambia, to trading networks (for example, fishing, charcoal). More destabilising was very pronounced mobility and more unpredictable mobility linked to transience (people moving in and out or through), porous boundaries, location near international borders or as an entry point to the city and people coming into the community at night to drink, socialise and/or engage in transactional sex.

### Class profile

Most communities had longer term lower middle-class residents in some areas, although usually fewer than lower income groups. In South Africa, these established middle-class residents were often located in central, core areas, with informal, burgeoning settlements on their boundaries. In Zambia, there was also a trend of better off middle-class residents moving into five communities.

### Substance abuse

There was pronounced substance abuse (alcohol, marijuana and, in South Africa, methamphetamine) across all communities and, although more evident at night and weekends, some residents would start drinking by mid-morning. Some communities were a hub for alcohol and recreational drugs, drawing outsiders in to engage in the use or trade of these substances. Tavern owners and other alcohol and drug traders wielded power and influence. In general, the more substance abuse in community, the less stable it was.

### Crime

Zambian communities had relatively less evident sexual violence and robbery than South Africa, although both were always a feature, more especially at night. In Zambia, crime was linked to clandestine activities including drugs, gambling, sex work and poaching. Crime and violence in South African communities were ubiquitous. Sexual violence was especially evident. While crime events were associated with moments of instability, in many places criminal activities were also closely linked to the local economy.

### Poverty

Poverty was evident in all communities, sometimes in pockets and sometimes more widespread. In South Africa, it was mitigated by child, old age and disability welfare grants. Established links with formal employment reduced poverty in some areas. Extreme poverty made places less stable.

## Responsiveness and resistance to intervention

In this analysis, we considered how people in a place responded to outside intervention, including HIV efforts, as either more responsive (collectively open) or more resistant (collectively more closed), building on the open-closed model of urban systems [16, 24].

### Open-closed assessment

Eight communities were assessed as open and connected at the core because people there demonstrated community leadership and action during disasters (for example, a cholera epidemic), strong leadership (political, ethnic, religious) that even if protectionist was open to change including HIV prevention, local organisational structures (for example, street committees in South Africa) and a strong shared communal history, pulling outsiders in because of their location, markets and openness. Eight communities were assessed as being too open and chaotic because of extremes of poverty, marginalisation, social tensions, mobility and population growth and/or they lacked leadership. Six were assessed as closed, demonstrating more resistance to outsiders, xenophobia and/or blaming tendencies, conservatism and protectionism. The caveat is that due to the variation within communities described earlier, there were sometimes a mix of open-closed. For example, SA18 was open to intervention but also in areas, demonstrated extreme mobility and population growth.

### Response to HIV initiatives

Response to HIV initiatives had different components that emerged out of the HIV literature and our own analysis (see Additional File 1, table of social factors and implications for HIV). These were history of HIV initiatives, alternative management of HIV, attitude to using HIV services (testing, treatment, prevention), the role of leadership and stigma. Examples are provided in Additional File 2.

### History of HIV initiatives

A stronger history of HIV initiatives was evident through one or more of the following: a legacy of innovative HIV/TB initiatives, including door-to-door testing campaigns, volunteer action and ART clubs; community health workers openly living with HIV; a history of HIV activism; key population initiatives (including those with Men who have Sex with Men (MSM), People With Disability (PWD), fisherfolk, sex workers); institutional HIV initiatives (police, prison, army, mines); and HIV services dovetailing with broader altruism and development. There was less history of HIV initiatives in other communities demonstrated by one or more of the following: absence of community health workers, limited evidence of NGO activities, no key population initiatives, middle-class residents utilising private health insurance and being left out of some initiatives due to location, class or ethnicity. Communities where there was a longer, richer history of HIV initiatives were generally more responsive.

### Other options to manage HIV

Prominent options for managing HIV with alternative (non-antiretroviral) treatment were more pronounced in some communities. Television evangelism was very popular in one community amongst the middle-class, faith healing and/or traditional medicine had a strong presence in several communities, and `immune boosters’ and other alternative medications and treatment (including herbs and other natural remedies) were evident in most communities. Communities where there was greater emphasis on alternatives were generally less responsive to health service-led interventions.

### Attitudes toward local health services

Willingness to use HIV services at the local health facility was notably enhanced by good relationships with health staff, flexibility toward ART supply for mobile clients living with HIV, integrated HIV services and a welcoming health facility with more discrete ART access. Resistance to use HIV services at the local health facility was more evident amongst middle-class and young people, and in some communities, there was a trend of residents living with HIV accessing services outside of their community. Health staff ‘speaking badly’ about clients or breaching confidentiality also put residents off accessing the facility. Overall, there was a widespread acceptance of HIV testing, and it became more unacceptable to not have been tested for HIV. In a few communities, there were rumours about health services delivering ‘fake’ or erroneous HIV test results. The better and more trusting the relationship with existing health services, the more responsive the community was.

### Established leadership

It was evident that if the established leadership and/or core of the community provided support to improvements in health including HIV interventions that this could counteract other forms of instability and resistance. For example, in Z9, cohesion about improvements in health across leadership and health conditions pushed back against other forms of instability.

### Stigma

Community level stigma data, both qualitative and quantitative, showed variability in stigma over time and across forms and sometimes across data sources. For the purpose of this analysis, high and/or increased HIV stigma was more closely aligned to resistance, and low and/or decreased stigma was linked to responsiveness. When placed alongside other community level dimensions, stigma patterns were usually more coherent, corresponding to wider socio-structural patterns [30, 31].

### Young people

Youth disillusionment was driven by limited education and livelihood options, a culture of blaming young people for HIV, high illiteracy, the limited presence of NGOs working with young people, high rates of teenage pregnancies and marriages and young people feeling let down by leadership. These were common across all communities and more pronounced in some. Generally, the happier and more engaged young people were, the more responsive the community was.

## Social factors across arms

We then classified each community in the HPTN 071 (PopART) trial according to the stability/instability and responsiveness/resistance dimensions grouped by arm and against the HIV incidence point estimate (Table 3). HIV incidence confidence intervals were wide since incidence estimates for individual communities were based on small numbers of events [2].

**Table 3:** Baseline HIV prevalence/incidence, stability/instability and responsiveness/resistance patterns across communities by HPTN 071 (PopART) study arm

Arm/site/triplet		Baseline HIV prevalence	HIV incidence*	Stability/instability	Responsiveness/resistance
**ARM A**					
Z2	Triplet 1	16.30% (14.2, 18.7)	1.64 (1.09, 2.38)	Mixed	Resistant
Z5	Triplet 2	17.40% (15.8, 19.2)	1.57 (1.08, 2.2)	Unstable	Mixed
Z8	Triplet 3	18.30% (16.5, 20.3)	1.36 (0.86, 2.04)	Stable	Resistant
Z10	Triplet 4	25.90% (23.9, 28)	1.93 (1.39, 2.62)	Unstable	Resistant
S14	Triplet 5	29.90% (28, 31.9)	2.36 (1.65, 3.27)	Mixed	Resistant
S16	Triplet 6	24.90% (23.1, 26.9)	1.43 (0.93, 2.1)	Unstable	Resistant
S19	Triplet 7	10.8%	0.5%	Unstable	Resistant
**ARM B**					
Z1	Triplet 1	17.0% (14.7, 19.4)	0.94 (0.57, 1.48)	Stable	Responsive
Z6	Triplet 2	23.4% (21.7, 25.1)	1.2 (0.8, 1.72)	Stable	Mixed
Z9	Triplet 3	21.5% (19.4, 23.7)	1.3 (0.81, 1.97)	Mixed	Mixed
Z11	Triplet 4	24.2% (22.1, 26.4)	1.13 (0.68, 1.76)	Mixed	Mixed
SA13	Triplet 5	29.2% (27.4, 31.1)	1.8 (1.24, 2.53)	Mixed	Resistant
SA18	Triplet 6	19.9% (18.3, 21.5)	1.24 (0.81, 1.82)	Mixed	Responsive
SA20	Triplet 7	9.9% (8.7, 11.3)	0.4 (0.19, 0.74)	Stable	Responsive
**ARM C**					
Z3	Triplet 1	21.9% (20, 23.9)	1.17 (0.75, 1.74)	Stable	Mixed
Z4	Triplet 2	18.1% (16.4, 20)	1.48 (1.02, 2.07)	Stable	Responsive
Z7	Triplet 3	19.6% (18, 21.4)	1.63 (1.1, 2.33)	Mixed	Resistant
Z12	Triplet 4	28.1% (25.5, 30.9)	2.39 (1.69, 3.29)	Unstable	Mixed
SA15	Triplet 5	28.0% (26, 30.1)	2.15 (1.43, 3.1)	Mixed	Resistant
SA17	Triplet 6	35.7% (33.6, 37.8)	2.31 (1.58, 3.27)	Unstable	Resistant
SA21	Triplet 7	3.0% (2.2, 4)	0.65 (0.36, 1.09)	Stable	Resistant

*HIV incidence confidence intervals were wide since incidence estimates for individual communities were based on small numbers of events

HIV incidence confidence intervals were wide since incidence estimates for individual communities were based on small numbers of events [2].

**Fig. 3:**
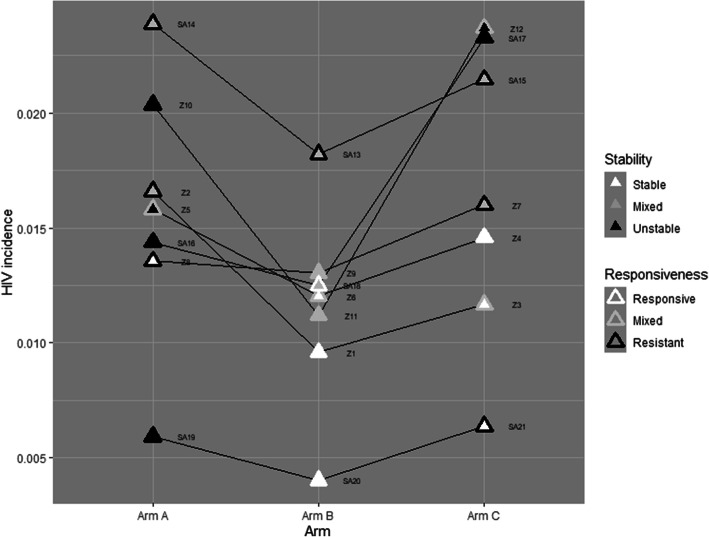
Community HIV incidence by arm and triplet. The three communities in each triplet (one randomised to each arm) are shown joined by a line and labelled. The symbol for each community illustrates the level of both stability (the inner shading) and responsiveness (the outer shading)

In general, it appeared that arm A communities were more resistant (only one of 7 was mixed responsive/resistant) and less stable (4 of 7 were unstable, 2 were mixed stable/unstable, one was stable). Arm B (intervention package initially without universal treatment) communities were more diverse in the overall pattern, but relative to arm A communities, arm B communities were more responsive and stable overall. Arm C (control) communities were diverse across these two social factors, more notably in Zambia. Figure 3 illustrates that in six of seven triplets, arm B communities had higher levels of both stability and responsiveness than their arm A counterpart; six of seven arm C communities had the same level of either responsiveness or stability as their arm B counterpart.

## Discussion

We distilled complex community dynamics into two social factors stability/instability and responsiveness/resistance. Our analysis provides an example of how community-level dynamics might create secular trends in disease incidence that are not typically measured in CRTs and, even when according to established trial practice, balance was achieved on the basis of demographic and disease epidemiology. It is a contribution to calls to give more detail on the role of community context in trial randomisation and outcomes [10, 13] and other contributions that highlight the role of social factors in HIV [17, 18, 29, 32] and HIV intervention implementation [12, 15, 33].

Similar to other evidence, the need to pay attention to the role of micro-epidemiology, the middle-class and the needs of young people is reiterated in our data [34, 35]. The presence of extreme social change, mobility, substance abuse, crime, the middle-class, poverty, higher levels of stigma and particular sexual behaviour patterns all make communities more vulnerable to HIV [17, 18, 30, 36]. Social cohesion, receptiveness to change, wider education options, better amenities, strong leadership, a robust local economy and institutional support [37, 38] emerge as boosting HIV prevention efforts. The momentum of broader HIV efforts, the diversity and type of urban systems and community socio-historical trajectories play a critical role in HIV prevention response and HIV incidence [21, 24].

German and Latkin [39] (p.19) argue that the role of social stability in health has been understudied. Our analysis underscores the pivotal role that stability plays in allowing HIV prevention to be taken up and the disruptiveness of instability. The sub-factors that fall under stability provide detail for this finding. For example, a local economy could be more robust or more stagnant with a robust local economy ensuring the daily presence of residents, supporting local HIV intervention delivery. Linked to this multi-dimensional and encompassing factor is response to outside intervention, with responsiveness supporting and resistance undermining navigation of HIV. Sub-factors again illustrate this concretely. For example, in relation to the profile of young people, the disillusionment of youth undermined HIV intervention and an enterprising and supported youth supported HIV intervention. We hypothesise that in the context of the PopART intervention, being assessed both stable and responsive indicated a promising moment in a community socio-historical trajectory to carry out an HIV prevention intervention. For a place to be unstable and resistant indicated a very challenging moment in a community socio-historical trajectory to carry out an HIV prevention intervention. We argue that based on our social science data and community variability in HIV prevalence and HIV incidence, the PopART intervention was important in the HIV incidence trajectory but the range and weight of social factors is likely to have reduced the ability of the intervention to dramatically reduce HIV in the way mathematical modelling predicted.

To accommodate the interaction of complex community context and HIV intervention, various approaches have been suggested. These include formative research [40], some degree of flexibility in implementation to interface with the synergy of research, time and context [33, 41], a Context and Implementation of Complex Intervention (CICI) framework [12], the importance of feasibility studies in CRTs [42], a closer integration of process evaluation and trial outcome data [25, 43], shifting to a more pragmatic trial design [44] and a conceptual framework (presented in this paper) on how to classify community characteristics in a variable to assist outcome interpretation.

## Strengths and limitations

Strengths of our analysis include (a) richness and variety of qualitative data collected in all 21 study communities and before, throughout and immediately after the study period, (b) that our data collection was pre-planned and specified to include an analysis of the influence of context on HIV incidence measured in the trial, (c) strong links to well-established social theory on the ‘place’, and the influence of social context, (d) a multi-disciplinary ‘social science’ team of analysts and interaction with all members of the trial leadership in interpreting the data and (e) appropriately presenting our findings as suggestive and hypothesis-generating.

Limitations of our analysis include that we collected fewer data from arms B and C—although even then the data set in these arms is atypically large for a study of this type. Some of the analysis was conducted after unblinding of the trial primary outcome which may mean that we have over-interpreted our data to ‘fit’ this outcome. We have mitigated this by explicitly stating that we are *not* able to make the claim that our results explain the outcome. Rather, we make a much more circumspect claim that our analysis suggests a plausible pathway to explaining secular trends and use the PopART outcomes as an example of how this could be done. A further limitation is that the stability and responsiveness continuums lose some of the complexity of community characteristics by reducing them to a limited set of sub-categories. Some of the ‘lower-level’ details on sub-components might be instructive without reduction to the two social factors, depending on the type of analysis required. We suggest that these classifications are applied to other settings with careful consideration of the aim of the analysis and that the characteristics are not simply used elsewhere as binary variables without careful consideration of local dynamics. Finally, in this analysis, we do not include much detail on sub-populations, sexual behaviour and stigma. These important areas of interest are to be included in other publications.

## Conclusion

In this paper, we set out an approach that enables local context to be rapidly, systematically and comparatively assessed and present two interdependent social factors that have implications for HIV. A crucial component of intervening to address the HIV epidemic is to know and work with the local context and systems in which the HIV epidemic unfolds. Working with or against these social factors more meaningfully and recognising the current trajectory of communities could, we argue, bolster the momentum of HIV effort and lead to more sustainable and greater impact on HIV incidence. This requires moving toward solutions that engage with broader development processes and stakeholders and by having a flexible intervention design that systematically addresses and rapidly assesses ‘what kind of place is this?’. Whilst recognising that not all population-based CRTs might have the resources and the disciplinary scope that PopART provided, our approach emphasises the importance of reflecting on the influence of place and patterns in urban systems that shape HIV and the response to HIV and proposes the meta-indicator framework as a strategy to organise community data. HIV incidence is on a trajectory (independent of intervention), and by paying attention to the features of communities, we can be more responsive to differences in trial implementation as well as results.

## Supplementary Information

**Additional file 1.**

**Additional file 2.**

## Data Availability

The datasets generated and/or analysed during the current study are not publicly available due to a trial undertaking to protect the identity of each of the communities but are available from the corresponding author on reasonable request.

## References

[1] UNAIDS. UNAIDS Data 2018 Geneva: UNAIDS; 2018 [Available from: https://www.unaids.org/sites/default/files/media_asset/unaids-data-2018_en.pdf.

[2] Hayes RJ, Donnell D, Floyd S, Mandla N, Bwalya J, Sabapathy K, Yang B, Phiri M, Schaap A, Eshleman SH, Piwowar-Manning E, Kosloff B, James A, Skalland T, Wilson E, Emel L, Macleod D, Dunbar R, Simwinga M, Makola N, Bond V, Hoddinott G, Moore A, Griffith S, Deshmane Sista N, Vermund SH, el-Sadr W, Burns DN, Hargreaves JR, Hauck K, Fraser C, Shanaube K, Bock P, Beyers N, Ayles H, Fidler S, HPTN 071 (PopART) Study Team (2019). Effect of universal testing and treatment on HIV incidence - HPTN 071 (PopART). N Engl J Med.

[3] Havlir DV, Balzer LB, Charlebois ED, Clark TD, Kwarisiima D, Ayieko J, Kabami J, Sang N, Liegler T, Chamie G, Camlin CS, Jain V, Kadede K, Atukunda M, Ruel T, Shade SB, Ssemmondo E, Byonanebye DM, Mwangwa F, Owaraganise A, Olilo W, Black D, Snyman K, Burger R, Getahun M, Achando J, Awuonda B, Nakato H, Kironde J, Okiror S, Thirumurthy H, Koss C, Brown L, Marquez C, Schwab J, Lavoy G, Plenty A, Mugoma Wafula E, Omanya P, Chen YH, Rooney JF, Bacon M, van der Laan M, Cohen CR, Bukusi E, Kamya MR, Petersen M (2019). HIV testing and treatment with the use of a community health approach in rural Africa. N Engl J Med.

[4] Makhema J, Wirth KE, Pretorius Holme M, Gaolathe T, Mmalane M, Kadima E, Chakalisa U, Bennett K, Leidner J, Manyake K, Mbikiwa AM, Simon SV, Letlhogile R, Mukokomani K, van Widenfelt E, Moyo S, Lebelonyane R, Alwano MG, Powis KM, Dryden-Peterson SL, Kgathi C, Novitsky V, Moore J, Bachanas P, Abrams W, Block L, el-Halabi S, Marukutira T, Mills LA, Sexton C, Raizes E, Gaseitsiwe S, Bussmann H, Okui L, John O, Shapiro RL, Pals S, Michael H, Roland M, DeGruttola V, Lei Q, Wang R, Tchetgen Tchetgen E, Essex M, Lockman S (2019). Universal testing, expanded treatment, and incidence of HIV infection in Botswana. N Engl J Med.

[5] Iwuji CC, Orne-Gliemann J, Larmarange J, Balestre E, Thiebaut R, Tanser F, Okesola N, Makowa T, Dreyer J, Herbst K, McGrath N, Bärnighausen T, Boyer S, de Oliveira T, Rekacewicz C, Bazin B, Newell ML, Pillay D, Dabis F, Bärnighausen T, Herbst K, Iwuji C, Makowa T, Naidu K, Newell ML, Okesola N, de Oliveira T, Pillay D, Rochat T, Tanser F, Viljoen J, Zuma T, McGrath N, Balestre E, Dabis F, Karcher S, Orne-Gliemann J, Plazy M, Prague M, Thiébaut R, Tiendrebeogo T, Boyer S, Donfouet H, Gosset A, March L, Protopopescu C, Spire B, Calmy A, Larmarange J, Inghels M, Diallo H, Calvez V, Derache A, Marcelin AG, Dray-Spira R, Lert F, el Farouki K, Lessells R, Freedberg K, Imrie J, Chaix ML, Newell C, Hontelez J, Bazin B, Rekacewicz C (2018). Universal test and treat and the HIV epidemic in rural South Africa: a phase 4, open-label, community cluster randomised trial. Lancet HIV.

[6] Boily M-C, Mâsse B, Alsallaq R, Padian NS, Eaton JW, Vesga JF (2012). HIV treatment as prevention: considerations in the design, conduct, and analysis of cluster randomized controlled trials of combination HIV prevention. PLoS Med.

[7] Gregson S, Adamson S, Papaya S, Mundondo J, Nyamukapa CA, Mason PR (2007). Impact and process evaluation of integrated community and clinic-based HIV-1 control: a cluster-randomised trial in eastern Zimbabwe. PLoS Med.

[8] Abdool Karim SS (2019). HIV-1 epidemic control — insights from test-and-treat trials. N Engl J Med.

[9] Storeng KT, Abimbola S, Balabanova D, McCoy D, Ridde V, Filippi V (2019). Action to protect the independence and integrity of global health research. BMJ Global Health.

[10] Wells M, Williams B, Treweek S, Coyle J, Taylor J (2012). Intervention description is not enough: evidence from an in-depth multiple case study on the untold role and impact of context in randomised controlled trials of seven complex interventions. Trials..

[11] Hawe P (2015). Minimal, negligible and negligent interventions. Soc Sci Med.

[12] Pfadenhauer LM, Gerhardus A, Mozygemba K, Lysdahl KB, Booth A, Hofmann B, Wahlster P, Polus S, Burns J, Brereton L, Rehfuess E (2017). Making sense of complexity in context and implementation: the context and implementation of complex interventions (CICI) framework. Implement Sci.

[13] Craig P, Gibson M, Campbell M, Popham F, Katikireddi SV (2018). Making the most of natural experiments: what can studies of the withdrawal of public health interventions offer?. Prev Med.

[14] Hanrahan CF, Schwartz SR, Mudavanhu M, West NS, Mutunga L, Keyser V, Bassett J, van Rie A (2019). The impact of community- versus clinic-based adherence clubs on loss from care and viral suppression for antiretroviral therapy patients: findings from a pragmatic randomized controlled trial in South Africa. PLoS Med.

[15] Sikazwe I, Eshun-Wilson I, Sikombe K, Czaicki N, Somwe P, Mody A, Simbeza S, Glidden DV, Chizema E, Mulenga LB, Padian N, Duncombe CJ, Bolton-Moore C, Beres LK, Holmes CB, Geng E (2019). Retention and viral suppression in a cohort of HIV patients on antiretroviral therapy in Zambia: regionally representative estimates using a multistage-sampling-based approach. PLoS Med.

[16] Wallman S, Bond V, Montouri MA, Vidali M, Conte RL (2011). The capability of places: methods for modelling community response to intrusion and change.

[17] Bates I, Fenton C, Gruber J, Lalloo D, Lara AM, Squire SB, Theobald S, Thomson R, Tolhurst R (2004). Vulnerability to malaria, tuberculosis, and HIV/AIDS infection and disease. Part 1: determinants operating at individual and household level. Lancet Infect Dis.

[18] Bates I, Fenton C, Gruber J, Lalloo D, Lara AM, Squire SB, Theobald S, Thomson R, Tolhurst R (2004). Vulnerability to malaria, tuberculosis, and HIV/AIDS infection and disease. Part II: determinants operating at environmental and institutional level. Lancet Infect Dis.

[19] Hayes R, Ayles H, Beyers N, Sabapathy K, Floyd S, Shanaube K, Bock P, Griffith S, Moore A, Watson-Jones D, Fraser C, Vermund SH, Fidler S, The HPTN 071 (PopART) Study Team (2014). HPTN 071 (PopART): rationale and design of a cluster-randomised trial of the population impact of an HIV combination prevention intervention including universal testing and treatment–a study protocol for a cluster randomised trial. Trials..

[20] Simwinga M, Bond V, Makola N, Hoddinott G, Belemu S, White R (2016). Implementing community engagement for combination prevention: lessons learnt from the first year of the HPTN 071 (PopART) community-randomized study. Curr HIV/AIDS Rep.

[21] Bond V, Chiti B, Hoddinott G, Reynolds L, Schaap A, Simuyaba M (2016). “The difference that makes a difference”: highlighting the role of variable contexts within an HIV Prevention Community Randomised Trial (HPTN 071/PopART) in 21 study communities in Zambia and South Africa. AIDS Care.

[22] Bond V, Ngwenya F, Thomas A, Simuyaba M, Hoddinott G, Fidler S, et al. Spinning Plates: Livelihood mobility, household responsibility and anti-retroviral treatment in an urban Zambian community during the HPTN 071 (PopART) study. J Int AIDS Soc. 2018; in press. 10.1002/jia2.25117 PMC6053474 30027643

[23] Bond V, Ngwenya F, Murray E, Ngwenya N, Viljoen L, Gumede D, Bwalya C, Mantantana J, Hoddinott G, Dodd PJ, Ayles H, Simwinga M, Wallman S, Seeley J (2019). Value and limitations of broad brush surveys used in community-randomized trials in Southern Africa. Qual Health Res.

[24] Wallman S. The diversity of diversity: implications of the form and process of localised urban systems. In: Second ENGIME (Economic Growth and Innovation in Multicultural Environments). London; 2003. Available at papers.ssm.com.

[25] Moore GF, Audrey S, Barker M, Bond L, Bonell C, Hardeman W, Moore L, O'Cathain A, Tinati T, Wight D, Baird J (2015). Process evaluation of complex interventions: Medical Research Council guidance. Br Med J.

[26] Hargreaves JR, Stangl A, Bond V, Hoddinott G, Krishnaratne S, Mathema H, Moyo M, Viljoen L, Brady L, Sievwright K, Horn L, Sabapathy K, Ayles H, Beyers N, Bock P, Fidler S, Griffith S, Seeley J, Hayes R, on Behalf of the HPTN 071 (PopART) study team (2016). HIV-related stigma and universal testing and treatment for HIV prevention and care: design of an implementation science evaluation nested in the HPTN 071 (PopART) cluster-randomized trial in Zambia and South Africa. Health Policy Plan.

[27] Shanaube K, Schaap A, Chaila MJ, Floyd S, Mackworth-Young C, Hoddinott G, Hayes R, Fidler S, Ayles H, HPTN 071 (PopART) Study Team (2017). Community intervention improves knowledge of HIV status of adolescents in Zambia: findings from HPTN 071-PopART for youth study. AIDS..

[28] Bond V, Hoddinott G, Viljoen L, Simuyaba M, Musheke M, Seeley J, on behalf of the HPTN071 (PopART) Study Team (2016). Good health and moral responsibility: key concepts underlying the interpretation of ‘treatment as prevention’ in 21 urban communities in South Africa and Zambia prior to rolling out universal HIV testing and treatment. AIDS Patient Care STDs.

[29] Milton S, Pliakas T, Hawkesworth S, Nanchahal K, Grundy C, Amuzu A, Casas JP, Lock K (2015). A qualitative geographical information systems approach to explore how older people over 70 years interact with and define their neighbourhood environment. Health Place.

[30] Hargreaves JR, Krishnaratne S, Mathema H, Lilleston PS, Sievwright K, Mandla N (2018). Individual and community-level risk factors for HIV stigma in 21 Zambian and South African communities: analysis of data from the HPTN071 (PopART) study. AIDS (London, England).

[31] Krishnaratne S, Bond V, Stangl A, Pliakas T, Mathema H, Lilleston P, Hoddinott G, Bock P, Ayles H, Fidler S, Hargreaves JR, on behalf of the HPTN 071 (PopART) Study Team (2020). Stigma and judgment toward people living with HIV and key population groups among three cadres of health workers in South Africa and Zambia: analysis of data from the HPTN 071 (PopART) trial. AIDS Patient Care STDs.

[32] Camlin CS, Seeley J (2018). Qualitative research on community experiences in large HIV research trials: what have we learned?. J Int AIDS Soc.

[33] Geng EH, Holmes CB (2019). Research to improve differentiated HIV service delivery interventions: learning to learn as we do. PLoS Med.

[34] Patton GC, Sawyer SM, Santelli JS, Ross DA, Afifi R, Allen NB, Arora M, Azzopardi P, Baldwin W, Bonell C, Kakuma R, Kennedy E, Mahon J, McGovern T, Mokdad AH, Patel V, Petroni S, Reavley N, Taiwo K, Waldfogel J, Wickremarathne D, Barroso C, Bhutta Z, Fatusi AO, Mattoo A, Diers J, Fang J, Ferguson J, Ssewamala F, Viner RM (2016). Our future: a Lancet commission on adolescent health and wellbeing. Lancet.

[35] Long D, Deane K (2015). Wealthy and healthy? New evidence on the relationship between wealth and HIV vulnerability in Tanzania. Rev Afr Polit Econ.

[36] Camlin CS, Cassels S, Seeley J. Bringing population mobility into focus to achieve HIV prevention goals. J Int AIDS Soc. 2018;21:(S4):e25136, 1–5. 10.1002/jia2.25136 PMC6053544 30027588

[37] Hargreaves JR, Glynn JR (2002). Educational attainment and HIV-1 infection in developing countries: a systematic review. Trop Med Int Health.

[38] Fonner VA, Kerrigan D, Mnisi Z, Ketende S, Kennedy CE, Baral S (2014). Social cohesion, social participation, and HIV related risk among female sex workers in Swaziland. PLoS One.

[39] German D, Latkin CA (2012). Social stability and health: exploring multidimensional social disadvantage. J Urban Health.

[40] Bond V, Hoddinott G, Musheke M, Viljoen L, Abrahams K, Chiti B (2013). Broad brush surveys of HIV prevention, treatment and care in 21 Zambian and South African communities to prepare for HPTN 071 (PopART).

[41] Singer M, Clair S (2003). Syndemics and public health: reconceptualizing disease in bio-social context. Med Anthropol Q.

[42] O’Cathain A, Hoddinott P, Lewin S, Thomas KJ, Young B, Adamson J, Jansen YJFM, Mills N, Moore G, Donovan JL (2015). Maximising the impact of qualitative research in feasibility studies for randomised controlled trials: guidance for researchers. Pilot Feasibility Stud.

[43] Oakley A, Strange V, Bonell C, Allen E, Stephenson J (2006). Process evaluation in randomised controlled trials of complex interventions. Br Med J.

[44] Davis K, Minckas N, Bond V, Clark C, Colbourn T, Drabble S, et al. Beyond interviews and focus groups: a framework for integrating innovative qualitative methods into randomised controlled trials of complex public health interventions. Trials. 2019;20(329):1–16. 10.1186/s13063-019-3439-8 PMC6555705 31171041

