# Supplementary material for: How ‘place’ matters for addressing the HIV epidemic: evidence from the HPTN 071 (PopART) cluster-randomised controlled trial in Zambia and South Africa
Source: Trials. 2021 Apr 6;22:251. doi: 10.1186/s13063-021-05198-5 (PMC8025534; doi:10.1186/s13063-021-05198-5)
Supplement: Supplementary file 1 — Additional file 1. [file 13063_2021_5198_MOESM1_ESM.docx]

**Supplementary File 1**

We outline 1) the theoretical underpinnings of our analysis; 2) the qualitative data sources; 3) the qualitative analysis steps.

1. **Theoretical Underpinnings of the Social Science Component in the Trial Design**

**Definitions of Stability, Responsiveness and Resistance**

The stability of a place is multi-dimensional and relates to livelihood, mobility, institutions, relationships and justice providing a steady and constant environment for local residents within which they can live and achieve balance and well-being in their life (1). Responsiveness relates to how a place responds to the needs of local residents (2). Resistance relates to a place opposing either dominant ideologies or the introduction of an entity or programme (3). These social factors are key to the analysis.

**Open/Closed Model**

Using this urban systems model, it has been shown that communities that are open and remain socially connected (heterogeneous with overlapping and open social networks) are more responsive to outside intervention, whilst communities that are too open and chaotic (heterogeneous without overlapping social networks) or too closed (homogenous and tight social networks) are either less able to respond to intervention or will not allow interventions (4, 5). The model has informed the social science component of HPTN 071 (PopART) from the outset and played a role in this analysis and findings. The disciplinary origins of this approach are broad and include social anthropology, sociology, economics, architecture and public health.

1. **Qualitative Data Sources – Activities and Methods**

The first social science research activity carried out in all communities used a Broad Brush Survey (BBS) approach (6). In HPTN 071 (PopART), the BBS fieldwork was carried out in 2012-2013 concurrent with a rapid population census and community engagement processes and prior to implementation of the trial intervention and enrolment of the primary outcome evaluation cohort. The sequence of methods used in the BBS were: group discussions with health committees; transect walks spiralling out from the health facility to community boundaries; structured observations of the health facility, entry/exit points, transport depots, water points, recreational and commercial spaces (for example, video clubs, bars, hair salons, markets, churches), and additional night and weekend observations; group discussions with men and women in different age groups and from different geographical zones; and key informant interviews with people knowledgeable about HIV. Participants were also asked an open question about “what kind of place is this?” to gain a local understanding of community characteristics. All activities in the BBS were conducted using semi-structured observation sheets and interactive open-ended discussion guides. Interviews and group discussion were audio recorded and photos taken throughout activities.

The second main activity was to document community response to the trial, encompassing intervention and research implementation, community response and participation. We also responded to unanticipated events (for example, a cholera epidemic), changes in HIV national guidelines (a shift to universal treatment) and intervention challenges (for example, slow linkage to HIV care). Labelled as “Story of the Trial”, this activity was carried out from 2014 to 2018, stretching from the start of the intervention (January 2014) to a period following the end of the intervention (December 2017). Qualitative methods included mainly structured observations of the intervention, the research and community engagement at the health facility and in the community, group discussions and in-depth interviews with intervention and/or research household members. In the non-intervention (arm C) communities, data collection was limited to observations of the research implementation, the health facility, community engagement and any events of relevance (for example, national guideline changes). Again, semi-structured observation sheets were used to record observations and open-ended interactive discussion guides to structure discussions, Researchers audio-recorded interactions with community members and took photos of all research activities. After field activities were completed, researchers completed reflection documents to note any additional observations from discussions. A centralised data management strategy across countries was used to enter, collate, and review data for quality and themes.

The third research activity in all communities was linked to another ancillary study PopART for Young people (P-ART-Y) that assessed the uptake of universal testing and treatment among young people (7). In 2015-2016, the social science team carried out structured observations of young people, focusing on gathering places (for example, schools, bars, markets etc.) and activities in these places, narratives about young people and their knowledge of HIV. Informal conversations with young people and older adult residents were conducted alongside the observations. In addition, a survey of all stakeholders involved in HIV services for young people and present in the communities was carried out, using an open-ended questionnaire that focused on type of services provided and successes and challenges. Rapid analysis was initially conducted to produce community specific short reports that were used for intervention planning. During the intervention period (2016-2017), structured observations were conducted of the intervention, including the interaction between young people and trial community health workers (in households), health workers (at the health facility), schools and other stakeholders (in the community).

For all three activities, following the completion of the analysis of each, we shared and discussed our findings with the 21 communities. For BBS, the sharing and discussion of the findings took place in HIV and trial stakeholder and community forums and helped decisions about intervention, research and community engagement. For the story of the trial, the findings were shared and discussed at district dissemination meetings as part of sharing all trial process findings. For P-ART-Y, the findings were discussed with communities and stakeholders to refine and determine the design of the intervention. All of these helped validate the characterization of communities.

**Qualitative Data Collection: team composition, training, and data quality assurance**

The teams responsible for data collection in both Zambia and South Africa were all local social scientists (from a range of social science disciplines) and trained and experienced in the field of qualitative health research. In South Africa, the field teams for all research activities included women and men who were fluent in English and either Afrikaans or Xhosa (local languages spoken in the Western Cape province) while in Zambia, researchers spoke English, Bemba, Tonga and Nyanja. Data were collected in teams, both to ensure data quality and for safety concerns in South Africa. In Zambia, a core team of three graduate social scientists were retained throughout the trial and each was responsible for four Zambian communities, and assisted by community-based research assistants, collected data for all components of the study from 2013 to 2019. In South Africa, there was some change in social science field staff during the trial period, although the two lead social scientists remained the same. Due to the closer proximity to the community in South Africa, all social scientists and research assistants worked across communities, although language strengths determined where they spent most of their time collecting data. In both countries, senior researchers provided practical and supervisory support.

Table 1: Social Science Teams in Zambia and South Africa

|  | **Zambia** | | | **South Africa** | | |
| --- | --- | --- | --- | --- | --- | --- |
|  | **BBS** | **SoT** | **P-ART-Y** | **BBS** | **SoT** | **P-ART-Y** |
| **Senior Researchers** | 3 | 2 | 2 | 2 | 2 | 2 |
| **Graduate Researchers** | 3 | 3 | 4 | 2 | 8 | 8 |
| **Research Assistants** | 8 | 8 | 8 | 2 | 14 | 14 |

Prior to all research activities conducted as part of the trial, a training package consisting of an interactive workshop on relevant materials, activities, data management, safety, ethics, and anticipated challenges were presented to in-country teams who were trained together in Zambia. At the multi-day workshops, team members would role-play all activities and discuss any concerns. Additionally, all team members were trained on Good Clinical Practice (GCP) and Human Subjects Protection (HSP). All data collection tools were piloted in one Zambian community with the whole team and, after feedback, amended as needed. During data collection, teams would receive refresher training and regular debrief sessions with senior researchers, both in group and one-on-one setting to discuss concerns, key events, or emerging findings.

All data were quality checked after collection. Hard copies were submitted to a central in-country data office and electronic copies saved on a password-protected computer. Data were sorted according to site, community and activity for ease of navigation. The data managers in South Africa and Zambia would conduct regular data audits to ensure that all data were stored accordingly. For analysis, all data were uploaded and managed using the software programme, ATLAS.ti.

**Data Analysis for BBS, Story of Trial and P-ART-Y**

For the BBS, there were two main analysis approaches: rapid analysis for applied outputs and more careful analysis for academic analyses. The rapid analysis was necessary to meet the demands of the trial and to ensure that local context influence trial research and intervention implementation and to support community engagement processes. Building on the debriefing meetings, social scientists wrote long narratives on each community, drawing on all BBS data sources and following a structured analysis template that was based on the meta-indicator framework and developed during the writing up the first long narrative from the first Zambian community where BBS was carried out. They reviewed all the data they had collected using the different research activities and summarised their findings. For example, the transect walk GPS readings, observation notes and photographs would be reviewed and then summarised. Any transcripts (FGD and IDIs) would be listened to and summarised. Each social scientist wrote up not more than four long community narratives. Once the long narratives were written and reviewed by lead social scientists in each country, short narratives were written up and reviewed and revised. The next step was to produce short narratives matrices on each community that split the findings according to research, intervention and community engagement needs. These were shared with all the field teams and community advisory boards. The short narrative reports and matrices were frequently used by the trial team and stakeholders throughout the trial. A BBS technical report for the funder summarised the findings across all 21 communities and was written by the first author with support from other social scientists. For this Place analysis, we mainly drew on the long narrative and short narrative BBS reports, the BBS technical report and the IDIs on community features with individual social scientists blind to the primary outcome results conducted for the Place analysis (see below). Once the BBS was rapidly written up, a coding framework was developed for the raw data. Then all data were coded. These coded data were and are drawn upon for other trial linked analyses led by or including social scientists.

For Story of the Trial data that focused on the intervention, research and community engagement implementation, data were not rapidly analysed unless there was a specific request from the trial team to look at particular issues. For example, low uptake by men or challenges with linkage to HIV care services. A coding framework was developed and all data were coded and used for focused analyses. For this Place analysis, one social scientist (blind to the primary outcome results) synthesised coded data onto responses to the PopART intervention (arms A and B) and responses to other HIV intervention (all arms) into a matrix that detailed each community.

The P-ART-Y BBS data followed a similar rapid analysis process to the main BBS to generate short narrative reports and matrices that were used to design the intervention. The raw data were then coded and used for focused analyses. For the Place analysis, a social scientist (blind to the primary outcome results) used the coded data to look at different features of adolescents in relation to where they gathered, HIV services and narratives about young people.

1. **Qualitative Data Analysis Steps on `the Place’**

In order to systematically synthesise qualitative community level data, we carried out a series of analysis steps that we detail below and in Figure 1 in the main paper. Some of these were carried out prior to knowledge of the primary outcome results, and others after it. The analysis after the unblinding focused on the social science data, and although the HIV outcomes were brought into the analysis process at every step (see templates), the analytical triangulation between two social factors and HIV incidence across trial arms only occurred after Step 4 was completed.

1. Meta-indicator Analysis (prior to primary outcome results)

We held an analysis workshop in September 2018 with team members who carried out the BBS in 2012-13 and had since collected a variety of the other data reported on above. We used the model of diverse urban systems that consists of four meta-indicators of infrastructure and population (visible and countable features), social organisation (relation of people to place), networks (relation of people to people) and community narratives (stories about the place) (1) The main objective of the workshop was to use our data on each community to qualitatively assess and score each community according to the relative presence or absence of a meta-indicator, reaching a final score for each community along a qualitative scale of a 0 to -3 (closed/homogenous) and 0 to +3 (open/heterogeneous). For example, entry/exit points are one component of the infrastructure and population meta indicator. Clear boundaries with few entry/exits would be scored as -3 to -1 (on the closed side of the continuum), and unclear boundaries with many entry/exits as +1 to +3 (on the open side of the continuum). This qualitative scoring involved synthesising social context detail, comparing one place with another by a group of social scientists who know the communities. The workshop was facilitated to first define the meta-indicators more finely to allow us to arrive at a score. For example, under social organisation, one component is population movement and the possibilities for population movement ranged from staying put to lots of people moving in lots of directions for many reasons. Secondly, we broke into in-country groups to draw up brief summaries of each meta-indicator for each community entered on a matrix. Finally, we scored the communities on each component (3 per indicator) of each of the four indicators. The theoretical range of the scale was -36 to +36 and the observed range for these communities was -33 (closed) to +27 (open).

1. Key Features relevant to HIV

The un-blinding of the trial results was on 17^th^ of December 2018 and the lead, second and senior author were part of a smaller trial team that learnt of the trial results before the results were presented at a conference (CROI 2019). Using the meta-indicators scoring did not reveal any clear pattern of the meta-indicators and HIV incidence outcomes across arms. We therefore decided to use a different approach. This has parallels with the trial modellers whose model projections did not turn out to be correct (8) and they then refitted model to see if there was a better agreement with the data. Drawing on the same data (collected prior to and during the trial implementation period) we identified features in the communities of significance to HIV based on literature. We identified six, namely: social history; the profile of local residents; local options for livelihood, recreation and treatment; mobility; stigma; and population knowledge about HIV. We used these six features to conduct in-depth individual interviews with in-country social scientists, about communities where they had conducted social science trial research. Three social scientists were also tasked with conducting a further synthesis of qualitative Story of the Trial, P-ART-Y and stigma data whilst still blind to the results. This process was completed by February 2019.

In March 2019, we then reviewed one Zambian triplet that had had an unexpected primary outcome result using a matrix that used the six features of significance, drawn from the interviews with social scientists, combined with HIV prevalence, incidence data and population numbers to generate an analysis approach and start identifying key contextual influences. We were aiming to abstract a limited number of aspects of social context that affected (enhanced or undermined) the ability of residents to navigate HIV.

1. Key influences – population profile, open/closed model, stability/instability, cross-cutting

This step allowed some keys influences to emerge. Firstly, the profile of the local population including the size, class, ethnicity and diversity. Secondly, the open-closed model according to community response to the trial interventions and open/closed features, with a distinction between open but connected and too open and chaotic (4-6). Thirdly, the stability and instability of a place. Fourthly, there were cross cutting influences in the form of the HIV profile of the community, sexual behaviour, stigma and young people.

Having determined the main influences, the lead author then went through all the data for each community, drawing on the sources listed earlier in methods and combined with the in-country social scientist interviews, the meta-indicator scoring for different components and the HIV prevalence and incidence measures, using a matrix for each of the four areas of key influences to condense data, and ending the sweep of all the data for each community with a short community summary. Communities were reviewed one by one within each Arm of the trial, allowing for a comparison first of communities within Arms and then across Arms once the analysis process was completed. Other social scientists were then asked to review and check the detail on each community that they knew well. Having generated these detailed community matrices, two key and interdependent social aspects were apparent as significant. These were stability/instability as the encompassing social factor and emanating from this responsiveness/resistance as significant to a community’s response to outside intervention including HIV. Our reasoning for reducing community context to binary categories was to be able to communicate these across disciplines, within a wider CRT audience and to allow them to work with quantitative outcomes.

Under step 3, we include a table summary of how these social factors directly relate to HIV.

1. Two social factors – stability/instability and responsiveness/resistance.

The lead author then generated a summary table of all communities organised into groups by Arms with key demographic, community narrative, HIV, stability/instability and responsive/resistant details, using the earlier matrices to arrive at this table (see Figure 1 and Figure 2 in main manuscript). This process allowed a qualitative assessment to be made for each community about whether a community was more stable or unstable, more responsive or resistant or a mix of both, with an explanation of how this assessment was arrived at. Again, the social factor assessment of each community was checked by other social science members. An example of community level data is provided in Table 3 in the main paper.

1. Triangulating social factor assessments with HIV incidence.

The final analysis step was to triangulate the two assessed social aspects (stable/unstable and responsive/resistant) of each community with community level HIV incidence outcomes and arms and to see if a pattern emerged. This step was only carried out once step 4 had been completed for all 21 communities.

Templates for the analysis (Steps 1 through 4) are included below. For Step 4, we include a community level data example to illustrate how we reached a decision about whether a community was stable/unstable or responsive/resistant or more a mix of both. Due to the risk of identifying communities, we had to remove some identifier detail.

**References**

1. German D, Latkin CA. Social stability and health: exploring multidimensional social disadvantage. Journal of Urban Health. 2012;89(1):19-35.

2. Goe WR, Noonan S. The sociology of community. In: Bryant CD, Peck DL, editors. 21st Century sociology: A reference handbook. California, USA: Sage; 2007. p. 455-64.

3. Hollander JA, Einwohner RL, editors. Conceptualizing resistance. Sociological forum; 2004: Springer.

4. Wallman S. The diversity of diversity: implications of the form and process of localised urban systems. Second ENGIME (Economic Growth and Innovation in Multicultural Environments); London2003.

5. Wallman S, Bond V, Montouri MA, Vidali M, Conte RL. The capability of places: methods for modelling community response to intrusion and change. London: Pluto Press; 2011.

6. Bond V, Ngwenya F, Murray E, Ngwenya N, Viljoen L, Gumede D, et al. Value and limitations of Broad Brush Surveys used in Community-Randomized Trials in Southern Africa. Qualitative Health Research. 2019;29(5):700-18.

7. Shanaube K, Schaap A, Chaila MJ, Floyd S, Mackworth-Young C, Hoddinott G, et al. Community intervention improves knowledge of HIV status of adolescents in Zambia: findings from HPTN 071-PopART for youth study. AIDS. 2017;31(3):S221-S32.

8. HIV Modelling Consortium Treatment as Prevention Editorial Writing Group. HIV treatment as prevention: models, data, and questions—towards evidence-based decision-making. PLoS Medicine. 2012;9(7).

Step 1: Meta-indicator summary template table (used at September 2019 analysis workshop) and meta-indicator scoring template

| **Meta Indicators** | | | | |
| --- | --- | --- | --- | --- |
|  | **Architectural Housing Options** | **Employment Work/Economic Options** | **Terrain (Physical Boundaries)** | **Population (Diversity, Ethnicity, Age, Class etc.)** |
| **Infrastructure & Population** | Community X  Community Y | Community X  Community Y | Community X  Community Y | Community X  Community Y |
|  | - *Description of housing available in Community X* - *Description of housing available in Community Y* | - *Description of work/ options available in Community X* - *Description of work/ options available in Community Y* | - *Description of physical boundaries in Community X* - *Description of physical boundaries in Community Y* | - *Description of population in Community X* - *Description of population in Community Y* |
| **Social Organisation** | **Distribution of categories of population across housing options** | **Distribution of categories of population across work options** | **Access to transport facilities and local services** | **Population movement in and out** |
|  | Community X  Community Y | Community X  Community Y | Community X  Community Y | Community X  Community Y |
|  | - *Description of population across housing in Community X* - *Description of population across housing in Community Y* | - *Description of population across work in Community X* - *Description of population across work in Community Y* | - *Description of transport/ facilities in Community X* - *Description of transport/ facilities in Community Y* | - *Description of population movement in Community X* - *Description of population movement in Community Y* |
| **Networks** | **Patterns of connection and relationship** | **Network spread: Extensive or intensive** | **Social Capital: Bonding or bridging** | **Network boundaries: Flexible or fixed?** |
|  | Community X  Community Y | Community X  Community Y | Community X  Community Y | Community X  Community Y |
|  | - *Description of networks in Community X* - *Description of networks in Community Y* | - *Description of networks Community X* - *Description of networks in Community Y* | - *Description of social capital in Community X* - *Description of social capital in Community Y* | - *Description of network boundaries in Community X* - *Description of network boundaries in Community Y* |
| **Narratives** | **Oral history of the place, (*including history of HIV)* Myths of origin & local style** | **Identification with the local above work or origin** | **Commitment to this place. Chosen? No plans to move?** | **Who is the butt of gossip? Who is blamed for disease & misfortune?** |
|  | Community X  Community Y | Community X  Community Y | Community X  Community Y | Community X  Community Y |
|  | - *Description of oral history in Community X* - *Description of oral history in Community Y* | - *Description of identification with local place in Community X* - *Description of identification with local place in Community Y* | - *Description of commitment to place in Community X* - *Description of commitment to place in Community Y* | - *Description of blame/ gossip in Community X* - *Description of blame/ gossip in Community Y* |

The tables below are used in conjunction (as one table in Excel) to determine the Total score per place. The communities were scored on each component (3 per indicator on a scale of -/+3) of each of the four indicators. The theoretical range of the scale was -36 to +36 and the observed range for these communities was -33 (closed) to +27 (open).

|  | **TOTAL** | **Infrastructure and population** | | | | | **Social organisation** | | | | |
| --- | --- | --- | --- | --- | --- | --- | --- | --- | --- | --- | --- |
| **Community X** | **0** |  |  |  |  | 0 |  |  |  |  | 0 |
| **Community Y** | **0** |  |  |  |  | 0 |  |  |  |  | 0 |
|  |  | **Architecture and housing options** | **Employment/ economic options** | **Terrain (physical boundaries)** | **Population diversity (age, class etc.)** | **Total** | **Distribution of people across housing** | **Distribution of people across job opportunities** | **Access to transport and local services** | **Population movement in and out** | **Total** |

|  | **Networks** | | | | | **Narratives** | | | | |  |
| --- | --- | --- | --- | --- | --- | --- | --- | --- | --- | --- | --- |
| **Community X** |  |  |  |  | 0 |  |  |  |  | 0 |  |
| **Community Y** |  |  |  |  | 0 |  |  |  |  | 0 |  |
|  | **Patterns of connection and relationships** | **Network spread (intensive/ extensive)** | **Social capital (bonding/ bridging)** | **Network boundaries (fixed/ flexible)** | **Total** | **Oral history of place (singular/ plural)** | **Identification with local over work/ origin** | **Commitment to this place (default/ chosen)** | **Singular or plural 'other'** | **Total** | **Cityscapes** |

Step 2: Key features relevant to HIV

| 1. **SOCIAL HISTORY** | **Response** (Community X) | **Reflections** |
| --- | --- | --- |
| History of HIV initiatives |  |  |
| Community Narratives – Open to Change |  |  |
| Community Narratives - Vulnerability |  |  |
| Popular Ideas & Belief Systems |  |  |
| Community Leadership & Engagement |  |  |
| Influential individuals & groups |  |  |
| Signs of Significant change 2014-2018 |  |  |
| Response to [intervention] over time |  |  |

| 1. **POPULATION PROFILE** | **Response** (Community X) | **Reflections** |
| --- | --- | --- |
| Young people |  |  |
| Middle Class |  |  |
| Hard to Reach Groups |  |  |
| Population Mix & Diversity |  |  |
| Sexual Networks |  |  |
| Other networks |  |  |
| Population density & size |  |  |

| 1. **LOCAL OPTIONS** | **Response** (Community X) | **Reflections** |
| --- | --- | --- |
| Livelihood Options |  |  |
| Informal/formal mix |  |  |
| Scope of trade & markets |  |  |
| Clandestine Activities |  |  |
| Recreational options |  |  |
| Treatment options |  |  |
| Any other options of influence |  |  |

| 1. **MOBILITY** | **Response** (Community X) | **Reflections** |
| --- | --- | --- |
| Degree of Mobility |  |  |
| Mobility patterns |  |  |
| Mobile residents |  |  |

| 1. **STIGMA** | **Response** | **Reflections** |
| --- | --- | --- |
| Degree over time |  |  |
| Health Facility stigma |  |  |
| Stigmatised Groups |  |  |
| Stigma linked to CHiPs |  |  |

| 1. **KNOWLEDGE ABOUT HIV** | **Response** | **Reflections** |
| --- | --- | --- |
| Scope of HIV prevention options |  |  |
| Response/awareness of UTT & TasP |  |  |
| Nicknames for ART & LWH |  |  |
| Response to ART & linkage to care over time |  |  |
| Alternative options for HIV management |  |  |

Step 3: Key influences

| 1. **Population profile** | | | |
| --- | --- | --- | --- |
| **Communities** | **Age** | **Class** | **Ethnicity** |
| **Community X**  Population Diversity: 2  Population: 23,991  Adults over 18: 11,995 | *Age distribution of population* | Description of class of population groups in different areas within the community | Population ethnic composition |
| **Community Y**  Population Diversity: 1  Population: 29,293  Adults over 18: 4,925 | *Age distribution of population* | Description of class of population groups in different areas within the community | Population ethnic composition |

| 1. **Open/Closed Model** | | | |
| --- | --- | --- | --- |
| **Community response to [intervention]** | **Closed** | **Open but connected at the Core** | **‘Too’ open, chaotic** |
| **Community X – [Open/closed score]**  *Narrative description of community response to [intervention], including response to intervention components; knowledge of the study/intervention; mobilisations; appreciation or resistance towards intervention over time;* response from different groups in the community.  *Reflections on predicted response to research, intervention and community engagement: Challenges and facilitators.* | *Components that received ‘closed’ (negative) scores from the open/closed model and a short descriptive narrative.* | *Components that received ‘open’ (positive) scores from the open/closed model and a short descriptive narrative.* | *Descriptive narrative of components that are too open/chaotic.* |

| 1. **Stability** | | | | |
| --- | --- | --- | --- | --- |
| **Community** | **Stability** | | **Some stability** | **Instability** |
| Community X | *Descriptive narrative related to education, employment opportunities, components of community cohesiveness, community density; infrastructure services; economic prospects.* | | *Descriptive narrative related to education, employment opportunities, components of community cohesiveness, community density; infrastructure services; economic prospects.* | *Descriptive narrative related to education, employment opportunities, components of community cohesiveness, community density; infrastructure services; economic prospects.* |
| 1. **Stigma** | | | | |
| **Communities** | | **Stigma profile** | | |
| Community X  Open/closed score: xx | | *Blaming: [score received]*  *Qualitative narrative description, including from in-depth interviews, and observations at health facilities.*  *Quantitative measurements of types of stigma over time; including internalised stigma; experienced stigma; health care setting stigma; resilience etc.*  *Summary of stigma: Qualitative data and quantitative data summaries.* | | |

| 1. **Crosscutting themes** | | | | |
| --- | --- | --- | --- | --- |
| **Communities** | **HIV profile** | **Sexual Behaviour profile** | **Stigma profile** | **Young people profile** |
| *Community: X*  *Intervention Arm:*  *Triplet:*  *Open/closed score:* | *Baseline HIV prevalence:*  *HIV incidence outcome:*  *HIV initiatives:*  *Narrative description of historical access to HIV testing and treatment; presence of community health workers; neighbourhood health committees; NGOs; other HIV initiatives; health facility access; community health knowledge; traditional and/or faith healers.* | *Description of sexual behaviour; including from vulnerable groups* | *Blaming: [score]*  *Data from IDIs and observations at the local Health Facilities.*  *Quantitative:*  *Statistics on measured stigma, including anticipated, internalised, and experienced*  *Summary of stigma: Qualitative and quantitative data.* | *School options; substance abuse concerns; economic opportunities; attitudes towards health services.* |

| 1. **Community Summary** | | |
| --- | --- | --- |
| **Community X**  **[Receiving same intervention components]** | **Community Y**  **[Receiving same intervention components]** | **Community Z**  **[Receiving same intervention components]** |
| *Summary: Extended narrative description. Include components related to historical overview of community; changes in community structures over time (prior and during intervention); overview of class; employment; economic prospects; population; open/closed profile; leadership; community concerns, including crime and poverty; attitude towards and prospects of youth; patterns of stigma.*  *HIV-related narratives including history; response to the intervention; engagement of different community groups with the intervention components; response form the health system.* | *Summary: Extended narrative description. Include components related to historical overview of community; changes in community structures over time (prior and during intervention); overview of class; employment; economic prospects; population; open/closed profile; leadership; community concerns, including crime and poverty; attitude towards and prospects of youth; patterns of stigma.*  *HIV-related narratives including history; response to the intervention; engagement of different community groups with the intervention components; response form the health system.* | *Summary: Extended narrative description. Include components related to historical overview of community; changes in community structures over time (prior and during intervention); overview of class; employment; economic prospects; population; open/closed profile; leadership; community concerns, including crime and poverty; attitude towards and prospects of youth; patterns of stigma.*  *HIV-related narratives including history; response to the intervention; engagement of different community groups with the intervention components; response form the health system.* |

Table: Social Factors and Implications for HIV

| **COMMUNITY CONTEXT** | **IMPLICATIONS FOR HIV** |
| --- | --- |
| *Kind of trial community* | Micro-level differences in HIV epidemiology within trial community. |
| *HIV prevalence & incidence trajectory* | Historical HIV incidence trajectory may be increasing, plateaued or decreasing prior to and during the trial period. |
| ***Stability/Instability*** | **Stability enabled communities to better navigate HIV. Instability was disruptive to HIV.** |
| *More/less social cohesion* | Social cohesion built the ability of communities and sub-populations to manage HIV. |
| *Limited/extreme social change* | Extreme change made HIV control harder. Needed to be receptive to some change for intervention uptake. |
| *Local economy vibrant/stagnant* | Robust local economy ensured daily presence of residents, supporting local HIV intervention delivery. |
| *Education options more/less* | Wider local education options were protective against HIV, especially for girls. |
| *Better/worse water & sanitation* | If better, reduced vulnerability to & daily risks of other infectious diseases. Linked interventions enhanced local capacity to actively support other health initiatives. |
| *Better/worse housing* | Middle-class housing harder to reach door-to-door (physical & social barriers) & dwellers more absent. Very poor housing often more accessible, but offered little privacy, space, protection and dignity to manage HIV. |
| *Stronger/weaker institutional presence* | Strong institutional presence (even if protectionist) facilitated HIV initiatives by providing leadership & structures. |
| *Predictable/unpredictable mobility* | Pronounced and/or unpredictable mobility undermined contact with HIV services. |
| *Established/newer middle-class residents* | Established lower middle-class lent steadiness, education, shared history & leadership to HIV initiatives. Newer middle-class more detached, often accessing work, school and health care including HIV services outside community. |
| *More/less amenities within community* | More amenities provided constructive outlets & boosted community identity to address HIV. |
| *Substance abuse less/more pronounced*. | Substance use undermined accessing HIV services, proved disruptive in health facilities & was linked to transactional sex, sex work, sexual violence and intergenerational sex. |
| *Heightened/limited crime* | Heightened crime undermined HIV initiatives by limiting reach of interventions, perpetuating sexual violence & amplifying HIV risk. Community driven crime control supported other community action including against HIV. |
| *More/less poverty* | Extreme poverty made it harder to prioritise health and HIV and increased vulnerability to HIV. |
| ***Responsiveness/Resistance*** | **Responsiveness enabled communities to better navigate HIV interventions. Resistance was disruptive.** |
| *Open/Closed Profile* | Open yet connected at the core was more receptive to HIV intervention than closed or chaotically open. |
| *Response to HIV initiatives* | A stronger history & range of HIV initiatives, fewer alternative treatment options, better relationships with health facilities, strong leadership and, wider acceptance, responsiveness and inclusiveness boosted HIV management. |
| *Community Stigma* | Lower and/or decreased stigma was linked to more openness & responsiveness, and less targeted blaming for HIV. |
| *Profile of Young People* | Disillusionment of youth undermined HIV intervention. Enterprising & supported youth supported HIV intervention. |

Step 4: Social factors

| **RCT Arm &**  **Site** | **Demographics** | **Narratives** | **HIV** | **Stability/ Instability** | **Responsiveness/ Resistance** |
| --- | --- | --- | --- | --- | --- |
| **Template:** | | | | | |
| *Arm:*  *Community:*  *Triplet:* | *Population size:*  *Population mix: Class, ethnicity, population composition* | *Description of: Community narratives of the place; the health facility/services; NGOs; economic/education prospects; ‘feel’ of the community; etc.* | *Baseline HIV Prevalence:*  *HIV incidence:*  *Descriptive narrative of history and response to HIV interventions; stigma; sexual networks; etc.* | *More/less stable/unstable:*  *Narrative description of aspects related to stability/instability from Steps 1 through 3.* | *More/less responsive/resistant:*  *Narrative description of aspects related to responsiveness/ resistance from Steps 1 through 3.* |
| **Detailed Example:** | | | | | |
| **ARM:**  Community:  Triplet: | Population Size: 28,830 (19,220 >18)  Population mix: Mix of middle class (established, some more affluent, intergenerational shared houses) & lower SES (informal densely populated settlements). Ethnically dominated by one group. Other ethnic group residents moving into the informal parts of the community. Some foreign (African) nationals also reside in the community. | Community originally one ethnic/racial group. Nicer place to live than surrounding areas: (some gardens attached to houses); opportunities are better (room to expand & move in, less overwhelming); closer to town centre. The clinic was rebuilt as a day hospital in the formal area. Public hospital & TB hospital nearby. Active NGOs for youth & two HIV NGOs providing options for accessing ART. In the past, food parcels for handed out to PLHIV. ART has an informal market (recreational). However, the community is weighted down by lack of prospects & being overlooked (described themselves as lost sheep). Distrust in political parties. Described as “steady but boring”. Concerns about sustainability of PopART. | PCO HIV Prevalence: 9.9%  HIV incidence: 0.4%  Navigating HIV is enhanced by being more trusting of HIV interventions & health facilities & social support; and undermined by stigma, crime (gangs), intergenerational sex & transactional patterns, more limited HIV service options, sub-group resistance (middle class, transgender persons, MSM, young people) & alternative treatment options. | More stable.  Stability: Older, steady community at heart (with older money) & people coming in are sometimes connected to long-term residents. Feels established. Area of formal housing shared by generations. Predictable daily & seasonal mobility. Some developments (new hospital + roads), amenities (library, sports fields).  Instability: Lacks leadership, informal racial segregation between two groups, narrow economic options (farm, factory, tourism, but limited). Shacks built in yards of small houses. Informal areas growing on fringes & more vulnerable to crime, organized crime (gang, drug dealing, robberies), violence (robberies), rubbish recycling, shebeens & clubs. | More responsive.  Responsive: Formal areas keep local crime activity in check. Church plays an important role. Community is keen for new initiatives & very open to PopART. Residents are trusting of CHiPs (& grateful for work, ‘knowing’ the CHiPs is seen as a good thing). There were more requests for HIV tests over time. 7 NGOs are working with young people. There are mixed stigma patterns (upswing in PLWH but other forms reduce over time or stay steady).  Resistant: Closed community that feels neglected. Coloured middle-class long-term families at centre. Resistance from middle class with private medical aid. Linkage to care slow (stigma, health worker attitude, queues, other social responsibilities). Traditional medicine & faith healing influential. Crime is an issue. Gangs, drugs, alcohol linked to transactional sex. Transgender people & MSM are hard to reach.  Young people feel ‘stuck’ and ‘bored’ and crime is a way out. |
