# Supplementary material for: How ‘place’ matters for addressing the HIV epidemic: evidence from the HPTN 071 (PopART) cluster-randomised controlled trial in Zambia and South Africa
Source: Trials. 2021 Apr 6;22:251. doi: 10.1186/s13063-021-05198-5 (PMC8025534; doi:10.1186/s13063-021-05198-5)
Supplement: Supplementary file 2 — Additional file 2. [file 13063_2021_5198_MOESM2_ESM.docx]

**Supplementary File 2**

This supplementary file presents two tables that illustrate community examples of the two interdependent social factors, namely stability/instability and responsiveness and resistance.

**Table 1: Stability and Instability Features**

| **STABILITY** | **INSTABILITY** |
| --- | --- |
| *Social cohesion:*  In the community of Z6, there was strong sense of social cohesion and social capital organized around families associated with the fish trade. In addition, leadership associated with ruling party was prevalent. | *Clear Social Divisions:*  In SA19 in South Africa, there was a rapid rise of new informal settlements during the PopART intervention. New neighbourhoods were added to the outskirts of existing areas. There were tensions, including, at times, racial tension between established residents and incoming residents. |
| *Limited social change:*  Z1 in Zambia was a stable community with only limited movement of middle-class residents into poorer areas. It was a connected, predictable and contained community with relatively strong economic and political networks, local education and health options and a strong sense of community pride. | *Extreme social change (recent/near past):*  Prior to the PopART trial, the community of SA14 in South Africa went through a phase of extreme social change. Previously, the area was mostly informal, rapidly constructed housing. These housing structures were then replaced by formal government subsidised housing and planned neighbourhood. |
| *Local economy vibrant:*  In the community of SA13 in South Africa, the train station was a major transport hub and residents were able to easily reach employment opportunities in the city, at factories or on nearby farms. | *Local economy stagnant:*  In the community of Z10 in Zambia, the closing of local light industries meant that there were job losses and few opportunities for residents to thrive economically. |
| *Education options present:*  In Z3, community members had strong educational aspirations and access to wider options, outside of the confines of their neighbourhood. There were several secondary schools within the community. | *Limited education options:*  There were no secondary schools within the community of Z9 in Zambia. As such, children who wanted to attend secondary school had to travel to other, neighbouring areas. |
| *Better water & sanitation:*  In Z4, Zambia, the neighbourhood had planned sewerage, sanitation services and working water facilities. | *Poor water & sanitation:*  In SA16 in South Africa, water and sanitation services were markedly restricted. Some local residents resorted to the contentious “bucket systems” because of the lack of safe, usable toilets. Resentment towards the bucket system led to public protests. |
| *Better housing:*  In the community of SA14 in South Africa, there was a core area with planned, formal government-subsidised housing with access to electricity and water. | *Poor housing:*  In Z9 in Zambia, shared family housing with a family all sharing one room, commonly referred to as ‘mudadada’, was prevalent. |
| *Strong institutional presence (through government, politics or employment)*  Residents of Z4 in Zambia had strong links with the ruling party. In addition, the community has historical links with the council. | *Weak institutional presence*  SA17 was a relatively new community where services were just being developed and less visible and public protests about services were rising. |
| *Predictable mobility:*  The community of SA20 in South Africa was located close to farming areas. As such, there was predictable seasonal mobility associated with farming (grapes, deciduous fruits), especially during the summer months. | *Unpredictable mobility and/or extreme mobility:*  Community Z11 was located on the international border of Zambia and on the major transport routes. This meant that there were cross-border traders constantly moving in and out the community. |
| *Older/established middle-class residents:*  Z2 in Zambia was a stable community with long term residents. Many of these residents were part of an established lower middle-class working in the formal employment sector. | *New middle-class residents:*  In Z7, Zambia, new middle-class residents were moving in with few commitments to local community. |
| *Amenities within community:*  Just prior to the implementation of the HPTN 071 (PopART) trial, a new formal shopping centre that included a major grocery chain store was built in the community of SA14 in South Africa. | *Limited amenities within community*  In SA13 in South Africa, there were limited amenities across community. Aside from the local government clinic, community members had to travel to reach resources. |
| *Substance abuse less pronounced/present*  Compared to other communities in the trial, the community of Z3 in Zambia had fewer bars and limited marijuana smoking. | *Pronounced substance abuse:*  In SA 19 in South Africa, the area was considered to be a hub of alcohol and drug use. There were influential gangs supplying and distributing drugs, including methamphetamine and marijuana, to young people in particular. Alcohol abuse was widespread and prominent during weekends. |
| *Subdued poverty*  Poverty in the community of Z2 in Zambia was moderated because of established employment options in the formal market and in the government and mining sectors in the area. | *Glaring poverty (very dominant)*  Z5 in Zambia was marked by extreme poverty and strong class divisions. There were high levels of unemployment in the area. |
| *Limited crime*  In Z3, in Zambia, community members and stakeholders considered it to be a ‘safe place’ with few crimes and little violence. | *Heightened crime:*  In South Africa, the community of SA19 had high levels of criminal activity. In particular, there were active gangs engaging in often violent crimes. Violence against women, including sexual assault, was often described. Muggings, break-ins and assaults were common. |

**Table 2: Responsiveness and Resistance Features**

| **RESPONSIVENESS** | **RESISTANCE** |
| --- | --- |
| **OPEN-CLOSED PROFILE** | |
| *Open and socially connected*  The community of Z8 in Zambia demonstrated strong community leadership and action during a cholera epidemic. | *Too open, chaotic*  In SA16, South Africa, extreme mobility linked to employment and population growth meant that the community was often perceived as chaotic.  *Closed:*  The community of Z3 in Zambia was often described as conservative and ‘closed to outsiders’. |
| ***RESPONSE TO HIV INITIATIVES*** | |
| *Stronger history of HIV initiatives:*  In Z9, Zambia, there was a striking legacy of innovative HIV/ TB initiatives, including door-to-door testing campaigns and volunteer action. | *Less evident history of HIV initiatives:*  In SA19 in South Africa, residents who were more affluent tended to make use of private health insurance or medical aid and were less likely to take up the door-to-door services from the trial community health workers. |
| *Alternative HIV management much more subdued and less evident:*  In SA14, Traditional healers liaise with local health facilities to ensure that their clients were accessing and adhering to ART. | *Prominent options for alternative HIV management:*  In Z7, community members engaged in faith healing and traditional medicine. In addition, immune boosters, alternative medications and alternative treatment (including herbs and natural remedies) were often used. |
| *Willingness to use HIV clinic services:*  The neighbour health committee and the clinic strategy of using sensitive layout to accommodate the privacy of PLHIV meant that residents in Z4 in Zambia were willing to use the services at the local clinic. | *Resistance to HIV clinic services:*  In SA16, there was a mistrust of and extreme resistance towards the local health facility. This escalated and in May 2019, the health facility was burnt down by arsonists. |
| *Open to take up HIV testing & treatment:*  In Z1, from the beginning of the trial, most residents were keen to test for HIV and to have door-to-door testing services and responded enthusiastically to the offer of HIV self-testing later in the trial. | *Resistance to HIV testing and treatment:*  In SA13 in South Africa, community members raised concerns about ‘fake’ HIV results, including rumours of false positive results. Because of this mistrust, some community members were hesitant to test for HIV. |
| *Open to HIV prevention:*  In Z9, in Zambia, the needs of people living with disabilities were acknowledged and overall, the community was very receptive towards  HIV prevention efforts. | *Resistance to HIV prevention:*  In some communities, for instance SA13, SA14, SA15, SA16, SA17, and SA18 in South Africa, voluntary medical male circumcision was extremely unpopular due to ideological and practical conflicts with understandings of traditional circumcision practiced by Xhosa residents. |
| *Open to PopART intervention:*  In Z5, the community was open to outsiders and new ideas and happy to have the PopART intervention. | *Resistant to PopART intervention:*  In SA19, there were concerns about the confidentiality of PopART staff, fuelled by rumours that the trial community health workers were disclosing the HIV status of PLHIV in the community. |
| *Less stigma:*  In Z3 in Zambia stigma across different forms started high but, over time, as the study progressed, decreased for most forms. | *More pronounced stigma:*  In Z2, community members expressed concerns about being seen at the local health facility accessing HIV services, and inadvertently, having their status revealed. |
| **PROFILE OF YOUNG PEOPLE** | |
| *Youth more enterprising & supported:*  In Z1 in Zambia it was notable that young men were enterprising and ran small businesses. In addition, in Z1, there was an active support group for adolescents living with HIV and their parents. | *Youth disillusionment dominant:*  In Z2, Zambia, school leavers struggled to find work despite the middle-class profile in the community and the social support available to this group. |
